# The gut microbiome as a biomarker and modifiable risk factor in Lynch Syndrome

**DOI:** 10.1007/s10689-026-00540-9

**Published:** 2026-03-05

**Authors:** Verona Sarena Colaco, Annemarie Boleij

**Affiliations:** Department of Pathology, Radboud Institute for Medical Innovation, Radboudumc, Geert Grooteplein-Zuid 10, 6525 GA Nijmegen, The Netherlands

## Abstract

Lynch Syndrome (LS) is the most prevalent hereditary colorectal cancer syndrome, driven by germline mutations in DNA mismatch repair genes. Despite intensive colonoscopy surveillance, cancer risk among LS carriers remains highly variable, suggesting additional modifiers beyond genetics. Emerging evidence implicates the gut microbiome as a potential biomarker and modifiable risk factor in LS-associated carcinogenesis. This review synthesizes current findings on taxonomic and functional microbiome alterations in LS carriers, highlighting early dysbiosis characterized by depletion of butyrate-producing taxa and enrichment of virulent species such as *pks*+ *Escherichia coli*, *Fusobacterium nucleatum*, and enterotoxigenic *Bacteroides fragilis*. These oncogenic microbes promote DNA damage, inflammation and epithelial hyperproliferation in the mismatch repair deficient context, accelerating tumorigenesis. Functional signatures such as colibactin genotoxicity appear more predictive than taxonomic diversity. However, methodological heterogeneity, small cohorts and lack of longitudinal data limit biomarker validation. Finally, we outline future research that should integrate multi-omics, spatial profiling and genotype-stratified designs to identify clinically actionable microbial signatures. Understanding microbiome and host interactions in LS could assist in improved risk stratification beyond current standard surveillance guidelines.

## Introduction

How informative is the gut microbiome in predicting cancer risk in genetically predisposed individuals? Cancer risk in individuals with hereditary predisposition is influenced by complex interplay of phenotypic variations, genetic factors and environmental influences including the gut microbiome. While the gut microbiota has been extensively studied in sporadic colorectal cancer (CRC), its involvement in hereditary cancers such as Lynch Syndrome (LS) remains largely unexplored [1].

The gut microbiome is a vast ecosystem of 10–100 trillion symbiotic microbial cells, predominantly bacteria, that inhabit the human gastrointestinal tract. This diverse community is essential for health, assisting in nutrient metabolism and immune regulation. However, when microbial composition and function are disrupted, it can trigger chronic inflammation and alter host metabolism to promote tumorigenesis [2]. The bacteria driver-passenger model offers a conceptual framework for microbial shifts seen in CRC, where bacteria drivers initiate carcinogenesis by the acquisition of cancer hallmarks, while opportunistic passenger bacteria favour the subsequent altered pro-carcinogenic microenvironment [3]. Pathogenic bacteria and their metabolites can induce DNA damage, produce reactive oxygen species (ROS), stimulate cell proliferation and create a pro-inflammatory niche. These oncogenic microbes further accelerate disease progression by impairing anti-tumour immune responses, enabling malignant cells to evade immune surveillance and promote metastasis through epithelial-to-mesenchymal transition [4, 5]. In contrast, other commensal bacteria exert protective effects by reducing inflammation, inducing apoptosis and producing beneficial metabolites, such as butyrate which supports epithelial barrier integrity [6, 7]. But how do these contrasting pro- and anti-carcinogenic properties manifest in individuals with LS?

LS carriers harbour heterozygous germline variants in DNA mismatch repair (MMR) genes, conferring a markedly increased lifetime risk (30−70%) of early-onset CRC, endometrial cancer and other malignancies [8, 9]. LS-associated tumours usually exhibit a MMR-deficient (dMMR) molecular phenotype and high microsatellite instability (MSI-H), a hypermutation phenotype accelerating cancer progression compared to sporadic cases [10, 11]. The primary CRC precursor lesion in LS is the colonic conventional adenoma. However, recent research suggests that other precursor pathways exist, which could circumvent the traditional adenoma stage and develop through molecular changes in normal colonic crypts (aberrant crypt foci) [12–14]. The discovery of the MMR crypt foci and their frequent occurrence in LS have shed light on how tumours in LS form, however, not all precursor lesions in LS show dMMR and only few dMMR crypt foci develop into cancer, underscoring the diversity in molecular patterns contributing to carcinogenesis in LS. How the microbiome interacts with these different pathways to cancer is largely unknown.

Current guidelines recommend biannual surveillance colonoscopies to detect and remove precancerous lesions [15]. Yet, even among LS carriers with identical MMR gene variants, cancer risk varies significantly [16], suggesting that non-genetic modifiers, such as the gut microbiome, may play a critical role. Could the gut microbiome amplify the mutational burden in dMMR cells and directly influence cancer risk in LS carriers? Given its established association with sporadic CRC, this possibility warrants urgent investigation.

In this review, we summarize current evidence for involvement of the gut microbiome in LS-associated carcinogenesis, highlight knowledge gaps and discuss future directions. Understanding whether the gut microbiome can serve as a biomarker for identifying early carcinogenesis in asymptomatic high-risk LS carriers could transform cancer prevention strategies.

## Microbiome composition between LS carriers and controls

To determine whether the gut microbiome can serve as an early indicator of carcinogenesis in LS carriers, it is critical to first understand baseline differences between LS carriers and individuals without LS. A commonly assessed metric is taxonomic alpha diversity, which reflects species richness within a microbial community. However, findings across studies have been inconsistent when comparing LS carriers, including individuals with and without colorectal neoplasia, against non-LS controls.

For example, one study using shotgun metagenomic sequencing on faecal samples reported that LS carriers demonstrated lower species-level alpha diversity (Shannon-Wiener Index) compared to a non-LS sporadic cohort, aligning with the generalized model of dysbiosis [17] (Table 1). In contrast, another study using 16S rRNA gene sequencing (V4 region) on faecal samples observed significantly higher alpha diversity (Inverse Simpson Index) in LS carriers relative to non-LS controls [18]. Conversely, a family-based design with environmentally matched spouse controls applied 16S rRNA gene sequencing (V3-V4 region) to faecal samples and observed no significant differences in alpha diversity (Chao1, Observed Features and Simpson Diversity) between LS patients with CRC and their partners [19]. Similarly, a study of a culturally homogeneous cohort, in which all LS carriers shared the same *MSH2* variant, detected no significant differences in overall bacterial abundance or alpha diversity (Chao1 and Shannon Index) compared to non-LS family members [20]. This analysis was based on shotgun metagenomic profiling of faecal samples. Taken together, the highly controlled studies using environmentally matched spouse controls or genetically and culturally homogeneous cohorts, did not observe significant differences in alpha diversity between LS carriers and non-LS controls. This suggests that variability observed in other cohorts likely reflects environmental or genetic confounding rather than a LS-specific microbial signature. The strong environmental influence observed in LS cohorts using spouse controls or environmentally matched relatives is consistent with findings from large population-based microbiome studies in individuals without LS. Previous population-based microbiome studies and twin research have shown shared environmental influences such as diet, lifestyle and cohabitation substantially influence the gut microbiome composition, often exceeding variation contributed by host genetic factors [21, 22]. This highlights the importance of controlling environmental factors when identifying LS‑specific microbial signatures.

**Table 1:** Explanation of microbiome methodologies and metrics

Explanation of microbiome methodologies and metrics		
Sequencing methodologies	16S rRNA sequencing	This method targets the 16S ribosomal RNA gene which contains highly conserved and hypervariable regions (V1–V9) using specific primers, followed by PCR amplification and sequencing. Regions often used in microbiome research are V3-V4, V4, or full length 16S. It is limited in resolution beyond the genus level and does not capture functional genes [52].
	Shotgun metagenomics	This method involves fragmenting all DNA in a sample and sequencing the fragments without targeting any specific gene. This approach captures the entire genomic content of all organisms present, enabling high resolution taxonomic identification (species or strain level) and functional profiling (genes, pathways and metabolic potentials) [53].
Alpha diversity metrics (Within-sample diversity)	Shannon index (Shannon–Wiener Index)	A diversity measure capturing richness (the number of unique taxa) and evenness (how evenly taxa are distributed) in a community. Higher values indicate greater diversity [54].
	Chao1	An estimator of taxonomic richness that predicts the total number of unique taxa in a community, strongly weighted toward rare taxa [19, 20].
	Observed features	A count of the number of taxa detected in a sample (Operational Taxonomic Units (OTUs) or Amplicon Sequence Variants (ASVs)). It measures raw richness without estimating undetected taxa or accounting for evenness [23, 54].
	Inverse simpson index	Measures both richness and evenness but is more sensitive to dominant taxa. Higher values indicate more evenly distributed taxa and higher diversity [18].
Beta diversity metrics (Between-sample diversity)	Bray-curtis dissimilarity	Evaluates differences in taxonomic composition based on abundance between samples. It ranges from 0 (identical) to 1 (completely different). Frequently visualized using Principal Coordinate Analysis (PCoA) [18, 55].
	UniFrac distance	A phylogeny-based distance metric that considers whether taxa in two samples share evolutionary lineages. Can be weighted (abundance based) or unweighted (presence/absence) [56].
	Adonis analysis (PERMANOVA)	A statistical test applied to distance matrices (e.g., Bray Curtis, UniFrac) to determine whether differences in microbial community structure between groups are statistically significant [23, 57].

Another commonly assessed taxonomic metric is beta diversity, which assesses the variation in microbial communities between different samples. Similar to the conflicting findings observed for alpha diversity, the results regarding beta diversity differences between LS carriers and controls have been inconsistent across studies. Several studies, analysed faecal samples using shotgun metagenomics or 16S rRNA gene sequencing and reported no significant difference in overall beta diversity between LS carriers with and without colorectal neoplasia compared to controls [17, 19, 20]. Principal Coordinate Analysis was performed to visualise these microbial communities, and the Bray-Curtis dissimilarity metric was employed to assess variations in taxonomic composition. However, a study identified significant difference in beta diversity (Weighted UniFrac) between LS and non-LS control using 16S rRNA gene sequencing (V3-V4 region) to faecal and oral samples, with findings validated by Adonis analysis [23]. Similarly, significant global structural differences (Bray-Curtis dissimilarity) in the microbiome between LS carriers and non-LS controls were observed on faecal samples using 16S rRNA gene sequencing (V4 region) [18].

Despite the inconsistencies, several studies have identified recurrent taxonomic patterns associated with the LS status and the progression to CRC. Across multiple cohorts, faecal samples of LS patients showed a depletion of the phylum Firmicutes, specifically impacting protective butyrate-producing species such as *Faecalibacterium*, *Ruminococcaceae* and *Lachnospiraceae*. Simultaneously, there was a significant increase in the phyla Bacteroidetes and Proteobacteria compared to controls, particularly *Escherichia* [18, 20, 23, 24]. This microbial signature (increase Proteobacteria/decreased Firmicutes) and high prevalence of *Faecalibacterium*-poor enterotypes observed in LS individuals has also been associated with sporadic CRC and inflammatory bowel disease [17, 23]. The observed compositional shifts indicated that the LS gut environment favours a pro-inflammatory state. This leads to a decrease in symbiotic taxa that produce beneficial short-chain fatty acids (SCFA) like butyrate, which are essential for maintaining the epithelial barrier integrity. In a family-based study, the lack of microbial differences between LS patients and their environmentally matched spouses indicates that these shared lifestyle factors are not the primary cause of the microbial shifts associated with the disease [19]. The microbial differences observed in LS carriers could be a consequence of the MMR germline defect or resulting from local inflammation.

These discrepancies in generalized diversity patterns likely reflect methodological heterogeneity and the influence of confounding variables that shape the microbial niche. Such variability underscores the need for standardized approaches and careful considerations of environmental and technical factors when interpreting microbiome data in LS research especially considering the overall smaller sample sizes compared to sporadic cohorts [25, 26].

### Microbial diversity within LS carriers

Beyond comparing LS carriers to non-LS controls, it is equally important to examine microbial differences within the LS population. One study reported significant differences in beta diversity between LS carriers and controls yet found no diversity differences among LS carriers when stratified by the presence or absence of colorectal neoplasia [18]. This suggests that the LS status itself may drive microbial differences, but diversity metrics alone are unlikely to serve as reliable biomarkers for disease progression within LS carriers. Similarly, another study detected no significant differences at the phylum or genus level between LS patients with and without LS-associated extraintestinal cancer, though interpretation was limited by a very small sample size (*n* = 8) [24]. Together, these findings point to a broad dysbiosis in the baseline microbiota of LS carriers but underscore its inadequacy as predictor of neoplastic progression or LS-associated cancer risk in these studies. Further, no significant differences were observed between the faecal microbiota of LS patients who developed colonic precancerous lesions compared to those who developed gynaecological cancers [27].

## Early microbial changes in precancerous lesions

Only limited studies focus on the role of the microbiome in LS-carcinogenesis in early lesions. So far, the genus *Desulfovibrio* involved in sulphide production was enriched in both faecal samples and mucosal biopsies of individuals with adenoma detected during their initial screening [28]. On the contrary, the typically abundant beneficial butyrate-producing taxa *Clostridiaceae* were depleted in LS carriers with baseline adenomas. This suggests loss of protective microbial functions in the gut and a shift towards a potentially pro-inflammatory microenvironment. Interestingly, network analyses demonstrated that LS individuals with precancerous lesions displayed a higher *Escherichia* to *Bifidobacterium* ratio [29]. Their microbial networks are characterized by an enrichment of virulent taxa and a depletion of commensal symbionts. In contrast, LS patients without neoplasia tend to maintain symbiont centred networks. This shift corresponds to a transition from mutualistic to pathogenic functional profiles as carcinogenesis proceeds, suggesting that specific microbial signatures, rather than generalized diversity might hold greater promise as biomarkers for disease progression. Overall, the microbial shift observed in early lesions of LS carriers resembled those seen in later-stage CRC [28]. This suggests that dysbiosis in LS begins early in carcinogenesis.

## Microbial functional signatures that influence LS phenotype

Taxonomic composition and diversity metrics are limited in predicting neoplastic risk within the LS populations. Therefore, identifying the specific functional mechanisms by which the altered microbial community initiates carcinogenesis in the dMMR environment is more informative than establishing correlations in taxonomic composition (Table 2).

**Table 2:** Key microbial signatures in Lynch Syndrome associated colorectal cancer

Key microbial signature	Association with LS phenotype	Proposed functional mechanism in dMMR	Evidence level	Reference
*Escherichia*: *Bifidobacterium* Ratio	Higher ratio in asymptomatic LS carriers with precancerous lesions.	Network shift indicating loss of anti-inflammatory symbionts and protective functions such as butyrate production.	Human faecal samples for network inference.	[29]
Enrichment of oral-derived genera	Enriched in LS CRC tumour microbiota compared to controls	Oral–gut axis translocation, biofilm formation on colonic mucosa, metabolic cross-feeding that sustains hypoxic, pro-inflammatory niche	Human faecal samples and tumour tissue	[18, 34]
*Virulence Factors*				
*pks*+ *Escherichia coli*	Enriched intratumorally in LS-CRC, LS-adenoma and normal colonic adjacent mucosa. Predicts metachronous colorectal neoplasia risk in LS carriers (especially *MLH1* carriers).	Colibactin induces DNA double-strand breaks and accelerates mutagenesis in dMMR cells.	Human FFPE CRC, adenoma and normal colonic mucosa tissue samples.	[30, 31]
Enterotoxigenic *Bacteroides fragilis*	Identified in LS-adenomas and LS-CRC.	*Bacteroides fragilis* toxin disrupts epithelial barrier and promotes inflammation.	Human FFPE CRC and adenoma samples.	[30, 31, 46]
*Fusobacterium nucleatum*	Enriched distinctly in LS-CRC, not in LS tumour-free or LS-adenoma. Associated with advanced disease stage.	Fap2 adhesion protein binds to CRC glycans (Gal-GalNAc), promotes tumour colonization and facilitates immune suppression.	Human faecal samples and FFPE tumour tissue.	[17, 30, 31]
*Metabolic shifts*				
Catabolism of Amino Acids	LS-CRC gut microbiota exhibits increased functional capacity for lysine and arginine metabolism.	Supports epithelial turnover.	Human faecal samples for metagenomics and metabolomics.	[17]
Polyamine Biosynthesis	Elevated functional capacity in tumour-free LS carriers & LS-CRC.	Stimulates epithelial proliferation and DNA replication stress.	Human faecal samples for metagenomics and metabolomics.	[17]
Bile Acid Deconjugation	Increased functional activity in LS-CRC.	Increased production of secondary bile acids which induce oxidative stress and DNA damage.	Human faecal samples for metagenomics and metabolomics.	[17]
Reduced Carbohydrate Metabolism	Decreased amgK gene abundance in LS tumour tissue.	Disrupts peptidoglycan recycling.	Human FFPE CRC tumour tissue.	[34]
Flagellin (FliC)	FliC gene transcript was predictive of the interval development of preneoplastic colonic adenoma in LS.	Motility driven mucosal colonization and inflammation.	Human faecal samples for metatranscriptomics	[28]

A consistent finding across both LS and sporadic CRC is the enrichment of oncogenic or pro-inflammatory microbial virulence factors. Prior studies have noted the importance of colibactin, a secondary metabolite produced by *Escherichia coli* strains containing the polyketide synthases genomic island (*pks* + *E. coli*). This pathogenic genotoxin directly induces DNA double-strand breaks and interstrand cross-links in the host epithelial cells, thus contributing to genomic instability and tumorigenesis. The intratumoral presence of *pks* + *E. coli* is strongly correlated with a unique APC: c.835–8 A > G somatic mutation, indicating the specific DNA damage signature associated with colibactin [30]. This molecular association links colibactin producing *E. coli* to DNA damage in CRC, irrespective of hereditary or sporadic MMR deficiency. Moreover, the presence of intratumoral *pks* + *E. coli* in the initial CRC tumour was associated with an elevated long-term risk of developing metachronous CRC and subsequent neoplasia in LS patients [31]. This observation indicates that genotoxic damage imposed by colibactin accelerates mutagenesis in the already dMMR cells, thereby increasing the cancer recurrence risk.

Distinct faecal metabolites profiles such as polyamine biosynthesis pathways and reduced levels of amino acid derivatives were observed in LS subjects without adenoma or carcinoma [17]. These early alterations occur despite minimal changes in overall gut microbiota composition, suggesting that inherited MMR variants may influence host metabolic output and create an abnormal gut environment prior to major microbial shifts. Such changes could selectively favour the growth and pathogenicity of pro-carcinogenic species during tumour progression. Metatranscriptomic analyses further reveal that functional activity within the LS microbiome is highly concentrated. Only 17 core organisms account for approximately 70% of functional output, indicating that critical changes are driven by a small, influential subset [28]. Predictive transcripts associated with adenoma risk include shifts in flagellin contributors (*FliC* gene) and the establishment of an oxidative metabolic microenvironment [28]. The significance of *FliC* gene is corroborated across both genetic and sporadic CRC, highlighting a shared pathological mechanism related to the impaired gut epithelial integrity [32, 33]. Complementary metagenomic studies of LS tumour tissue demonstrate functional disruptions, such as a significant reduction in carbohydrate metabolism linked to decreased abundance of the *amgK* gene involved in peptidoglycan recycling [34]. Importantly, LS-CRC microbiota exhibits increased functional activity for lysine and arginine catabolism, alongside elevated polyamine biosynthesis and bile acid deconjugation [17]. These metabolic shifts likely promote epithelial hyperproliferation and oxidative stress, causing a pro-tumorigenic environment.

## Late microbial changes during LS-CRC

**Fig. 1:**
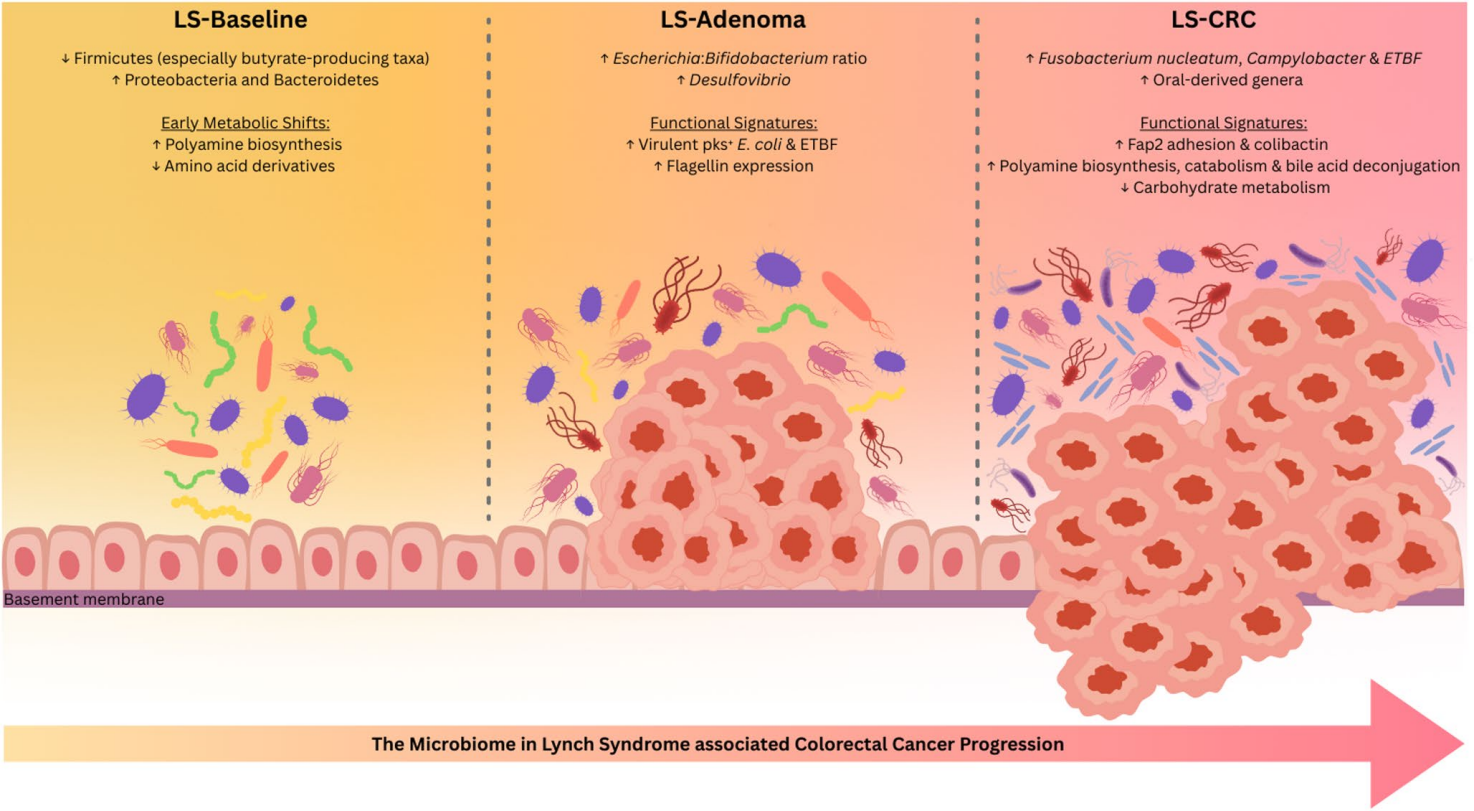
Microbiome shifts in LS patients at different stages of carcinogenesis

Building on these early alterations observed in precancerous lesions, the progression to CRC in LS involves distinct microbial changes (Fig. 1).

A consistent finding across multiple studies is the enrichment of *Fusobacterium nucleatum* (Fn) [17, 30, 31]. Fn species were significantly enriched in LS-CRC patients and were generally absent or minimally present in tumour-free or adenoma-bearing LS subjects. This suggests Fn is an indicator of established progression or late-stage CRC in LS, consistent with its enrichment in sporadic dMMR and CIMP-high MMR-proficient CRC subtypes [35]. Furthermore, LS-CRC individuals were over 19 times more likely to harbour intratumoral Fn compared to sporadic MMR-proficient CRC [31]. Beyond Fn, LS tumours exhibit enrichment of other taxa commonly associated with sporadic CRC, including *Campylobacter* and *Bacteroides fragilis*, as well as oral-derived genera such as *Veillonella*, *Streptococcus* and *Actinomyces*. This pattern suggests that LS tumour-associated microbiota share canonical CRC signatures despite differences in MMR origin, highlighting a potential oral–gut axis in LS neoplasia [18, 34]. Additionally, another study focused only on key oncogenic bacteria, including *pks* + *E.coli*, enterotoxigenic *Bacteroides fragilis* (ETBF) and Fn that were identified in 6.1%, 1.5% and 21% of the 66 adenomas [31]. These oncogenic bacteria were not only found intratumorally, but also in adjacent and non-adjacent normal colonic mucosa, implying widespread colonic colonization that may facilitate subsequent carcinogenesis.

Fn enrichment in LS-CRC was found to correlate with increased prevalence of its virulence factor Fap2, an adhesion protein that binds to Gal-GalNAc moieties highly expressed on CRC cells, facilitating Fn colonization within the tumour microenvironment [17]. Fn further promotes tumour survival by interacting with immune cell receptors, suppressing anti-tumour T-cell responses. Fn is associated with advanced disease stage, proximal tumour location and high levels of tumour-infiltrating lymphocytes in dMMR subtypes. Given its minimal presence in adenomas, Fn likely plays a more prominent role in cancer progression rather than initiation. Interestingly, compared to sporadic CRC, LS-CRC exhibits lower abundance of the virulence factor FadA, which mediates epithelial invasion and DNA damage [17]. Although multiple clades of Fn exist, strains significantly enriched in CRC tumours are members of Clade 2 (Fna C2). These strains are characterized by strong adhesion and immune-modulatory properties. In contrast, Clade 1 (Fna C1) species are predominantly oral commensals and are not as dominantly enriched in CRC tissue [36]. This distinction explains why particularly Fna C2 dominates within CRC tumours and contributes to disease progression.

In addition, evidence indicates specific pro-inflammatory Operational Taxonomic Units (OTUs) distinguish LS-CRC patients from LS carriers without cancer, suggesting that inflammation may contribute to CRC risk in LS. These enriched microbial species include *Streptococcus gallolyticus* and *Rothia dentocariosa*, as well as higher relative abundances of seven other *Streptococcus* species, *Enterococcus faecalis* and species belonging to the *Lactobacillus* genus [20].

## Insights from MMR-deficient mouse models

Distinct mouse models address the link between chronic inflammation in an dMMR environment and accelerated tumorigenesis [37]. *Msh2*^*loxP/loxP Vill−cre*^ crossed with *IL-10*^*−/−*^ double knockout mice were used to mimic LS with chronic inflammation, representing the combined effect of epithelial dMMR and IBD conditions. These site-specific proximal tumours exhibit bacterial invasion of crypt mucus, implicating mucosa-associated or biofilm forming communities in LS carcinogenesis. The inflammatory conditions result in the enrichment of genera such as *Escherichia-Shigella*, *Akkermansia*, *Bacteroides* and *Parabacteroides* and facilitates bacterial invasion directly into the mucus layer of tumour crypts [37]. Notably, *Akkermansia muciniphila* is generally considered beneficial in a symbiotic context, supporting gut barrier integrity through mucin utilization and SCFA production [38, 39]. In human IBD, however, its abundance is reduced, signalling a compromised mucosal barrier [40]. The observed enrichment of *Akkermansia* in this setting therefore suggests an inflammation-driven shift, whereby this species transitions from a commensal role to a pro-inflammatory under specific conditions.

The combined loss of *Msh2* and Transforming Growth Factor Beta Receptor 2 (*Tgfbr2*) was investigated using *Villin-Cre; Msh2*^*loxP/loxP*^;*Tgfbr2*^*loxP/loxP*^ mice to study the synergistic effects of dMMR and early loss of TGFBR2 signalling [41]. *TGFBR2* is a key tumour suppressor gene involved in the initiation of TGF-β signalling pathway and a frequent mutation target in dMMR CRC (up to 90% in LS and 70–80% in sporadic cases). The concurrent inactivation of MMR and *Tgfbr2* promotes inflammation-driven cancer (similar to human IBD-CRC) when coupled with DSS-induced colitis [41]. The resulting tumours displayed mutation signatures consistent with unrepaired replication errors and oxidative DNA lesion, while the damaged mucosa favours pathogenic taxa such as *Prevotella*, *Desulfovibrio*, and *Helicobacter.*

The critical role of the gut microbiota in dMMR carcinogenesis was further highlighted by the nearly complete elimination of intestinal tumour development observed when *Lgr5-CreERT2;Msh2*^*flox/−*^ mice were transferred from conventional housing into specific pathogen free (SPF) facility [42]. This mice model, known as *Msh2*-Lynch, introduces MMR deficiency specifically in intestinal stem cells, mimicking early crypt-level events in LS. LS tumorigenesis generally requires the acquisition of a somatic second hit in the remaining functional MMR allele in individuals carrying a germline pathogenic variant, resulting in complete MMR loss and the development of dMMR/MSI lesions [43]. The gut microbiome may act as an environmental driver of this process plausibly influence cancer risk by accelerating the occurrence of somatic inactivation through inflammation, oxidative stress (ROS) and the release of microbially derived genotoxins. Indeed, faecal microbiota transplantation from conventional housed mice to *Lgr5-CreERT2; Msh2*^*flox/−*^ mice restored epithelial hyperproliferation and increased mutation load (MSI) under SPF conditions [42]. This intervention study confirmed that conventional gut microbiota accelerates mutation accumulation and tumorigenesis in dMMR crypts. The pro-mutagenic effect was also associated with an elevated abundance of mucus-degrading taxa *Desulfovibrio* and *Akkermansia*, leading to mucus barrier loss and increased bacterial-epithelial contact in vulnerable MMR-deficient crypts [41]. The enrichment of mucus-degrading taxa was also observed in LS human patients [28].

Further, the link between ROS and chronic inflammation provides a strong mechanistic basis for aspirin chemoprevention in LS. Aspirin’s anti-inflammatory properties may help reduce ROS-mediated DNA damage, thereby lowering CRC risk in this high-risk population. Evidence for the role of oxidative stress comes from studies where antioxidants reduced oxidative DNA damage and polyp formation in colitis-associated cancer models using *IL10*^*−/−*^ mice to represent chronic intestinal inflammation and *Apc*^*Min/+*^ mice to modelling sporadic CRC with inflammatory triggers. However, the same antioxidant treatment and iNOS inhibitors failed to prevent CRC or polyp development in *Msh2*-deficient mice, including *Apc*^*Min/+*^;*Msh2*^*−/−*^ and *Apc*^*min/+*^*Msh2*^*flox/+*^ *Villin-Cre* mice [44]. These LS models introduce dMMR either systemically or specifically in intestinal epithelial cells, mimicking the genetic basis of LS. Although microbial-induced oxidative DNA damage (elevated 8-oxoguanine) does occur in dMMR colonocytes, the absence of functional MMR and the accumulation of replication errors dictates the high mutational burden characteristic of LS. Aspirin chemoprevention trials show significant CRC risk reduction in LS carriers [45], contrasting with the ineffectiveness of generalized antioxidant strategies. This discrepancy highlights the need for targeted interventions. Therapeutic strategies should focus on primary pro-mutagenic microbial mechanisms such as colibactin-induced genotoxicity or increased epithelial turnover rather than generalized anti-inflammatory approaches.

Collectively these findings indicate that LS carriers at high CRC risk harbour microbiota enriched in virulent, mucus-degrading taxa that compromise the epithelial barrier, promotes inflammation and induce oxidative DNA damage in dMMR crypts. This pro-inflammatory and genotoxic environment accelerates MSI-driven carcinogenesis. While oxidative stress contributes to this process, the absence of functional mismatch repair amplifies replication errors and mutational burden.

### Differences in microbiota between MMR-variants

The cancer risk and age of onset in LS carriers depends on the specific MMR variant. Despite this clinical heterogeneity, majority of current microbiome studies do not stratify based on MMR variant. This limits identification of mutation specific microbial signatures that may be critical for predicting risk. For example, the intratumoral presence of *pks* + *E. coli* in the initial CRC was associated with a 2.32-fold increased risk of developing subsequent metachronous colorectal neoplasia. However, this risk was significantly higher in *MLH1* pathogenic variant heterozygotes and not in *MSH2* carriers, indicating that the underlying MMR genotype influences the carcinogenic potential of specific bacteria [31]. This genotypic specificity may determine which microbial mechanisms most effectively promote neoplasia. Supporting this heterogeneity, LS pathogenic variants exhibit distinct taxonomic abundance profiles. For example, *Bifidobacterium*, a beneficial bacterium, was consistently depleted in carriers of *MLH1*, *MSH2* and *MSH6* variants. Notably, the MSH2 variant was uniquely linked to an increased abundance of *Actinomyces*, a bacterium involved in mucosal biofilm formation. In addition, both *MLH1* and *MSH2* carriers showed increased levels of the genera *Streptococcus* compared to the *PSM2* group [18]. Furthermore, *Bacteroides fragilis* abundance in LS-CRC biopsies has been associated with altered expression of MMR genes (*mlh1*, *msh2*, *msh6*), highlighting the need for further research into its potential role in modulating the MMR pathway [46]. Moreover, in a genetically homogeneous LS cohort with a shared *MSH2* variant, *Rothia dentocariosa* was the only species enriched in LS-CRC [20]. *Rothia dentocariosa* is a normal inhabitant of the oral microbiome and contributes to dental plaque biofilms, but its presence in LS-CRC is notable given that it is not considered major CRC-biomarker in sporadic cases [47, 48].

## Current barriers to reliable microbiome insights in Lynch Syndrome

Despite the increasing interest in the microbiome as a biomarker for LS-associated CRC, current research faces significant methodological and conceptual challenges. Most studies are underpowered due to small sample sizes, limiting statistical power and external validity [23, 24, 27, 49]. This often necessitates pooling all MMR gene variants rather than stratifying by individual variants, preventing identification of mutation-specific microbial signatures. Small cohorts also restrict adjustment for confounders such as age, diet, antibiotic use and colonoscopy frequency. Additionally, cohorts drawn from single centres or registries reduce generalizability across diverse lifestyles and healthcare systems.

Most studies lack longitudinal data and rely on cross-sectional sampling, which captures microbiota at a single time point, often after neoplasia diagnosis or surgery, hindering temporal or causal inference, and predictive biomarker development. Frequent colonoscopy surveillance and prior colorectal surgery, common in LS patients, further alter gut microbiota, introducing bias when comparing LS-CRC cases to controls. Resection surgery causes substantial loss of commensal bacteria and enrichment of opportunistic pathogens like Streptococcus, leading to functional reductions in SCFA levels, which can persist for a prolonged time [50].

Technical challenges further complicate biomarker identification efforts. Non-invasive faecal sampling limits insight into long-term microbial dynamics and fails to capture the specific communities at the mucosal interface or within microscopic precursor lesions, sites of critical host–microbe interactions influencing early neoplasia Metagenomic DNA analysis detects virulence genes but does not confirm in vivo expression or toxin activity, as most studies assess taxonomic potential rather than functional activity. High variability and methodological heterogeneity in sample handling, sequencing, and bioinformatics introduce batch effects and reduce reproducibility [6]. Many datasets rely on 16S rRNA profiling, which provides only genus-level resolution and obscures strain-specific differences. Finally, distinguishing LS-specific microbial signatures from those shared with sporadic CRC remains unresolved. LS tumours consistently exhibit enrichment of canonical CRC-associated taxa. It remains unclear which, if any, microbial features are truly LS-specific or if the timing and stability of these features is the key distinguishing factor.

## Next steps: from microbial signatures to clinical utility

To move toward clinical application, research must address these gaps through strategic design and advanced methodologies. Large, prospective, longitudinal cohorts are needed to identify microbial signatures associated with neoplasia onset, with standardized protocols for sample processing, and storage across sites. Current ongoing clinical trials such as the Metagenomic Evaluation of the Gut Microbiome in Patients with Lynch Syndrome and Other Hereditary Colonic Polyposis Syndromes (NCT02371135), represent initial steps in improving risk stratification for CRC in LS carriers [51]. Family-based designs incorporating spouses, non-mutation relatives, and mutation-positive relatives without neoplasia can help disentangle environmental influences from inherited risk [19, 20]. Stratifying participants by individual MMR variant will enable detection of genotype-specific microbial patterns.

Future studies should adopt integrated multi-omics approaches, combining metagenomics, metatranscriptomics, and metabolomics. This approach enables confirmation that virulence genes detected at the DNA level are actively expressed in vivo by pairing metatranscriptomics data with targeted toxin and metabolite assays. Given that metabolic shifts precede major taxonomic dysbiosis in tumour-free LS carriers, non-invasive biomarker detection should focus on early metabolic changes associated with the LS phenotype. Bulk faecal sampling risks overlooking relevant mucosa-associated biofilm communities that may play a critical role in LS-related carcinogenesis. The modest predictive power of stool metatranscriptomes for interval adenoma development (AUC ≈ 0.65) suggests that relevant microbial activity is highly localized, making faecal samples unsuitable for early-stage risk prediction [28]. Although mucosal biopsies and tumour tissue offer deeper insights into biofilms and virulence factors, such data remain scarce. Spatial context should be captured through multi-site mucosal biopsies paired with techniques such as fluorescence in situ hybridization (FISH) and spatial transcriptomics to visualize bacterial positioning and biofilm architecture. Predictive models using network inference and machine learning [18, 29] must be externally trained and validated across multiple independent LS cohorts and asses for clinical benefit.

For cost-effective clinical implementation, non-invasive faecal panels incorporating targeted assays for high-value biomarkers (e.g. *pks*+ island, virulence factors) should be evaluated alongside existing screening protocols. Finally, intervention studies and randomized controlled trials are essential to clarify the role of microbial communities in LS-related cancer development and test preventive strategies, including microbiome-targeted therapies targeting high-risk pathobiotions (e.g. antibiotics, bacteriophages) and dietary interventions such as high-fibre intake or targeted synbiotic formulations, aimed at restoring protective microbiota in the dMMR context.

## Conclusion

The gut microbiome has shown to play a critical role in LS. LS carriers exhibit early dysbiosis marked by depletion of butyrate-producing taxa and enrichment of mucus-degrading and virulent species such as *pks* + *E. coli*, Fn and ETBF. These microbes promote DNA damage, chronic inflammation and epithelial hyperproliferation in the dMMR epithelium, accelerating MSI driven carcinogenesis. Functional signatures including colibactin genotoxicity, polyamine biosynthesis and bile acid deconjugation appear more predictive of neoplasia risk than taxonomic diversity alone.

Evidence from human studies and dMMR mouse models underscores the causal role of microbiota in tumour initiation and progression. Importantly, the gut microbiome likely serves as a critical mediator between environmental exposures and the high phenotypic variability observed in LS. An adverse microbiome amplifies the mutation burden in dMMR prone crypts through inflammation, DNA damage and oxidative stress, thereby could be influencing the likelihood of acquisition of somatic second hits and cancer risk in LS carriers.

Despite these insights, current research is constrained by small sample sizes lacking genotype stratification, cross-sectional designs and methodological heterogeneity in sampling and sequencing. These constraints limit the translation of reliable, clinically actionable microbial biomarkers.

To overcome these barriers, future studies must prioritize: (1) longitudinal multi-omics studies to identify functional drivers of LS early carcinogenesis; (2) validating predictive models across independent LS cohorts to assess clinical utility; and (3) conducting intervention trials to evaluate preventive strategies including microbial modulation through dietary interventions and targeting high-risk microbial pathways. Bridging mechanistic insights with clinical application, microbiome research in LS has the potential to transform surveillance for high-risk individuals.

## Data Availability

No datasets were generated or analysed during the current study.
